# Trichotillomania and skin-picking disorder: associations with childhood maltreatment and the mediating role of depressive symptoms

**DOI:** 10.1186/s40359-026-05193-y

**Published:** 2026-07-20

**Authors:** Amalie Bramming Moen, Benjamin Hummelen, Erna Moen, Toril Dammen, Ingrid Melle, Diana Strand, Douglas Woods, Torun Grøtte

**Affiliations:** 1 Department of Psychology, NTNU, Trondheim, Norway; 2 Department of Research and Innovation, Division of Mental Health and Addiction, Oslo University Hospital, Oslo, Norway; 3 Outpatient Department of Obsessive-compulsive Spectrum Disorders, Division of Mental Health and Addiction, Oslo University Hospital, Oslo, Norway; 4 Institute of Clinical Medicine, Faculty of Medicine, University of Oslo, Oslo, Norway; 5 Section for Clinical Psychosis Research, Division of Mental Health and Addiction, Oslo University Hospital, Oslo, Norway; 6 Adult Psychiatry Unit, Division of Mental Health and Addiction, Institute of Clinical Medicine, University of Oslo, Oslo, Norway; 7 Department of Psychology, Loyola University, Chicago, USA; 8 Nidaros DPS, St.Olavs University Hospital, Trondheim, Norway

**Keywords:** Trichotillomania, Hair-pulling disorder, Skin-picking disorder, Childhood maltreatment, Depressive symptoms, Borderline personality disorder

## Abstract

**Background:**

Childhood maltreatment (CM) is a well-known risk factor for a wide range of adult mental disorders. However, little is known about the role of CM in trichotillomania (TTM) and skin-picking disorder (SPD). The current study therefore aimed to explore the relationship between CM and symptom severity in patients with TTM and SPD, as well as to compare their baseline CM levels with those of patients with borderline personality disorder (BPD) and a healthy control group.

**Methods:**

The Childhood Trauma Questionnaire-Short Form (CTQ-SF), along with validated measures of disorder-specific severity and depressive symptoms (e.g., MGH-HPS, SPRS, and PHQ-9), was administered to patients with TTM (*n* = 82), SPD (*n* = 75), BPD and subthreshold BPD (*n* = 123), and a healthy control group (*n* = 157) prior to treatment. Between-group differences in CM were explored with Kruskal Wallis tests, while disorder-specific associations in the TTM and SPD groups were examined through correlational and mediational analyses.

**Results:**

The TTM and SPD group reported higher levels of CM than the healthy control group, especially emotional abuse and emotional neglect. However, these levels were lower than in the BPD group. Several EMs were significantly positively associated with TTM and SPD severity, although the associations were more numerous and stronger in TTM than in SPD. Depressive symptoms fully mediated the relationship between emotional neglect and self-reported TTM severity and partially mediated the relationship between emotional abuse and self-reported TTM severity.

**Conclusion:**

These findings suggest that several forms of CM are prevalent in individuals with TTM and SPD. The results are also consistent with, though do not prove, an emotion-regulation model in which hair-pulling serves as a maladaptive coping strategy for depressive symptoms.

**Supplementary Information:**

The online version contains supplementary material available at https://doi.org/10.1186/s40359-026-05193-y.

Hair-pulling disorder, or trichotillomania (TTM), and skin-picking disorder (SPD) are characterized by recurrent and compulsive hair-pulling or skin-picking, leading to hair loss or skin lesions, and psychosocial impairment or clinically significant distress [1]. Hair-pulling most commonly affects the scalp but may also involve other areas such as eyebrows, eyelashes, and pubic region [2]. Skin-picking and scratching typically occur on the face, arms, fingers, and scalp [3]. Both disorders are classified as body-focused repetitive behavior disorders (BFRB) in the Diagnostic and Statistical Manual of Mental Disorders (DSM-5) and the International Classification of Diseases (ICD-11) [1, 4], under the chapter of obsessive-compulsive and related disorders. Epidemiological studies based on representative general population samples have found the lifetime prevalence of each disorder to be between 1 and 2% [5–7], and both TTM and SPD are highly comorbid with other mental disorders such as depression, anxiety, ADHD, and obsessive-compulsive disorder (OCD) [8–10].

The etiology of BFRBs is complex, involving both genetic and environmental factors [11, 12]. Childhood maltreatment (CM) might be an important environmental factor contributing to the development of these disorders. CM encompasses a range of adverse experiences, including emotional, physical, and sexual abuse, as well as emotional and physical neglect, that can have lasting negative effects on an individual’s mental health and overall well-being [13–16]. To date, there is some evidence indicating that individuals with TTM and SPD experience significantly more CM than healthy controls [17–19]. First, in a cross-sectional study by Lochner et al. [17], the Childhood Trauma Questionnaire (CTQ) [20] was administered to 36 patients with TTM, 74 patients with OCD, and 31 healthy controls. The two clinical groups reported significantly higher levels of CM compared to the healthy controls, with the largest differences in emotional neglect and physical abuse.

Second, in an online survey of 325 adults with pathological skin-picking [18], the skin-picking group reported significantly more CM (i.e., CTQ) than a case-matched healthy control group: 59.1% vs. 38.2%. The skin-picking group scored significantly higher than the control group on sexual abuse (*d* = 0.27), emotional neglect (*d* = 0.33), and emotional abuse (*d* = 0.49). A limitation of this study was that pathological skin-picking was identified through individuals’ scores on self-report measures (i.e., SPS-R and SPIS scores ≥ 7) rather than diagnostic interviews, which may lead to false positive cases [21].

Third, Yalçin et al. [19] compared 38 patients diagnosed with SPD with 40 healthy controls in terms of CMs, as assessed by the CTQ. Consistent with Lochner et al. [17] and Spitzer et al. [18], Yalçin et al. [19] found that the SPD patients scored significantly higher on childhood trauma than the healthy controls (*d* = 0.71), with the largest differences in emotional abuse (*d* = 0.76), emotional neglect (*d* = 0.73), and physical abuse (*d* = 0.52).

Taken together, these results indicate that CM is more strongly associated with TTM and SPD as compared with healthy controls, especially emotional abuse and emotional neglect. However, the samples sizes are generally small, and none of the studies have directly compared TTM and SPD within the same study. Moreover, given that CM is associated with virtually all forms of psychopathology [13–16], it is important to examine the extent to which TTM and SPD are associated with CM relative to other mental disorders. In this context, borderline personality disorder (BPD), which is among the disorders most strongly associated with CM [22–25], provides a useful benchmark for evaluating the strength of these associations in TTM and SPD. Furthermore, several studies have shown that individuals with subthreshold BPD (i.e., meeting four BPD criteria) have levels of psychopathology, functional impairment, and clinical burden comparable to those meeting full diagnostic criteria for BPD [26]. Therefore, both threshold and subthreshold BPD should be considered when examining the relationship between CM and these disorders.

Another important question in understanding the relationship between traumatic events and BFRBs is whether these events directly influence the severity of TTM or SPD or if their impact is mediated through other symptoms. Several authors [19, 27] have proposed an indirect pathway, suggesting that CM lead to negative affect (e.g., depressive symptoms, anxiety), with hair-pulling and skin-picking emerging as maladaptive coping strategies to regulate this distress. Consistent with this perspective, emerging evidence suggests that emotion dysregulation may represent a key transdiagnostic mechanism linking CM to BFRBs, with individuals engaging in these behaviors to alleviate or modulate aversive internal states [28].

Empirical support for this indirect pathway has been provided by studies such as Houghton et al. [27], who examined the impact of traumatic events in a sample of 85 adult TTM patients using a checklist of eight types of trauma, e.g., sexual assault, witnessing violence, physical assault, and accidental injury. They found that individuals with a history of trauma (52.9% of the sample) reported significantly higher levels of depressive symptoms (*d* = 0.57) and greater global TTM severity (*d* = 0.51) compared to the non-traumatized group. Although anxiety was initially hypothesized as a potential mediator, this pathway was not supported due to a non-significant association between anxiety and trauma. However, depressive symptoms emerged as a significant mediator in the relationship between traumatic experiences and hair-pulling severity. Specifically, the previously significant direct effect of trauma on TTM (B = 0.25, *p* < .05) became non-significant when depressive symptoms were included in the model, while the paths from trauma to depressive symptoms (B = 0.28, *p* < .05) and from depressive symptoms to TTM remained significant (B = 0.30, *p* < .01). It is important to note that Houghton et al. [27] examined major traumatic events without a specified developmental time frame, rather than focusing on CMs such as emotional abuse or neglect. This underscores the need for further research on the mediating role of depressive symptoms in the relationship between CMs and BFRB severity. In particular, studies involving individuals with SPD are warranted, given the limited research available on this population.

Building on this line of research, more recent studies further suggest that this relationship may involve a broader set of interconnected mechanisms, including attachment insecurity, alexithymia, and emotion regulation difficulties [29, 30]. In a sample of 105 patients with BFRBs and 90 healthy controls, CM (as measured by CTQ-28), depressive symptoms, and anxious attachment were identified as risk factors for the presence of BFRBs [29]. Moreover, externally-oriented thinking (i.e., a subscale of alexithymia) mediated the relationship between physical neglect and BFRB severity. These findings indicate that difficulties in identifying and processing emotions may represent an additional pathway linking CM to BFRB symptoms.

Consequently, the overall aim of this study was to examine the relationship between self-reported CM and the severity of TTM and SPD in a large clinical sample of individuals with TTM and SPD. First, we compared self-reported CM scores (as measured by the brief version of CTQ [31]) between the TTM and SPD groups and between these groups and patients with BPD and healthy controls. BPD was included as a clinical comparison group to assess whether exposure to CM is elevated in individuals with BFRBs not only relative to healthy controls, but also in comparison to another psychiatric disorder in which trauma is known to play a significant role [22–25]. Based on existing literature [18, 24], we hypothesized that individuals with TTM and SPD would score significantly higher on CM (particularly emotional abuse and emotional neglect) than the healthy control group, but lower than the BPD group.

Second, we explored the associations between five subtypes of CM and the severity of TTM and SPD. Based on previous studies [17–19, 27], we hypothesized that higher CM scores would be significantly positively associated with more severe symptoms of TTM and SPD, particularly for the subscales emotional abuse and emotional neglect. Moreover, we hypothesized that depressive symptoms would mediate the relationship between emotional abuse/emotional neglect and severity of TTM/SPD [18, 27]. Although we acknowledge the potential role of multiple pathways [29], depressive symptoms were selected as the mediator due to their well-established association with CM and TTM/SPD severity [27, 32], as well as prior findings supporting their mediating role between CM and TTM [27]. Furthermore, emotional abuse and emotional neglect were selected as predictors, as they have been most strongly and consistently associated with the severity of TTM and SPD in previous literature.

## Method

### Participants and procedure

The present study includes a total of 314 participants: 82 patients diagnosed with TTM, 75 patients diagnosed with SPD, 124 patients diagnosed with BPD, and 157 healthy controls. The BPD and healthy control groups were matched with the TTM and SPD groups in terms of age and gender.

Inclusion of TTM and SPD participants in the present study was based on the following inclusion criteria: diagnosed with TTM or SPD; age between 16 and 67; score ≥ 4 on the Clinical Global Impression Scale – Severity (CGI-S) [33], signed informed consent; able to understand and write Norwegian. In Norway, individuals aged 16 years and older can legally provide informed consent for research participation without parental or caregiver approval. Therefore, additional caregiver or parental consent was not required for underage participants in this study. Exclusion criteria were as follows: schizophrenia spectrum disorders; substance addiction; severe antisocial, schizotypal, or paranoid personality disorder; severe eating disorder; ongoing suicidal ideation; pervasive developmental disorders; mental retardation; and severe sequelae after brain injury. Patients with comorbid bipolar disorder and ADHD were included on the condition that their symptoms were acceptably regulated.

The inclusion of BPD participants in the current study was based on the following criteria: age between 18 and 67 years, ability to understand and read Norwegian, and meeting at least four diagnostic criteria for BPD. Exclusion criteria were the same as for the BFRB group except for two aspects, i.e., bipolar disorder type I was an exclusion criterion whereas ongoing suicidal ideation was not an exclusion criterion.

### Procedures TTM/SPD sample

The TTM and SPD patients were sampled from two Norwegian research projects: “Cognitive Behavioral Group Therapy for TTM” (registered at https://clinicaltrials.gov/ 21.04.2016, NCT02747771) and “Acceptance-enhanced habit reversal training in group format for patients with SPD”. Both projects are longitudinal, observational cohort studies of TTM and SPD patients who receive a combination of acceptance and commitment therapy and habit reversal training within regular mental health care. The TTM project started in 2012 and the SPD project in 2014, and both enrolled their final patient in April 2024. Both projects were approved by the Regional Committee for Medical Research Ethics (REK) in Norway (REK-IDs: 14368 and 26569) and conducted in accordance with the Declaration of Helsinki [34]. In 2022, REK approved the combination of data from the TTM and SPD projects, forming what is now referred to as “The Norwegian BFRB project”.

Between 2014 and 2020, patients were able to self-refer to the TTM and SPD projects. Initial screening for inclusion and exclusion criteria was conducted via a telephone interview with the leader of the BFRB treatment team, during which a preliminary diagnosis of either TTM or SPD was also assigned. From 2021 onward, patients were referred through a general outpatient clinic, where they first underwent an initial screening. A more comprehensive diagnostic evaluation was then carried out by a clinician with expertise in BFRBs. The clinicians involved represented different professional backgrounds (clinical psychologists, psychiatrists, and psychiatric nurses) and had all received training in BFRB diagnostics from the third (EM) or second last author (DW).

The diagnostic assessments included the Anxiety Disorders Interview Schedule for DSM-IV (used from 2012 to 2017) [35], the Mini International Neuropsychiatric Interview (used from 2018 to 2024) [36], the TTM DSM-IV diagnostic interview [37], and the Keuthen Diagnostic Interview for Skin Picking [38]. Although the presence of comorbid TTM in individuals with SPD, and vice versa, was not systematically assessed using a structured diagnostic interview, such an interview was conducted when there were indications of comorbid BFRBs. Consequently, three patients initially referred with SPD were reclassified as having TTM. Additionally, all participants rated the severity of any comorbid hair-pulling or skin-picking behavior on a 1–10 scale, with scores ≥ 5 considered indicative of clinically significant comorbidity. The validity of this self-report measure has not been formally established.

The present study used only cross-sectional, pre-treatment data. Of the 287 patients with TTM and SPD included in “The Norwegian BFRB project”, 157 completed the Childhood Trauma Questionnaire-Short Form (CTQ-SF) [31] and therefore constituted the final study sample. The majority of the missing data resulted from the exclusion of the CSQ-SF from the questionnaire package during the transition from paper to digital formats in 2022. There were no significant differences between the final study sample (*n* = 157) and the sample with missing CTQ-SF scores (*n* = 130) in terms of age (*t* = -1.56, *p* = .120), sex (χ^2^ = 2.15, *p* = .143), diagnosis (TTM vs. SPD) (χ^2^ = 1.67, *p* = .194), or symptom severity as measured by the Clinical Global Impression scale (*t* = -0.14 *p* = .890).

### Procedures BPD sample

Participants with BPD (meeting at least five BPD criteria; *n* = 92) and subthreshold BPD (meeting four BPD criteria; *n* = 31) were recruited within the context of two earlier research projects, i.e., “the Ulleval Personality Project” (*n* = 53) [39] and “the Norwegian Study of the Alternative Model for Personality Disorders” (*n* = 70) [40]. Symptom disorders were assessed using the Mini International Neuropsychiatric Interview [36]. Personality disorders, including BPD, were assessed using the Structured Clinical Interview for DSM-IV Axis II Personality Disorders [41] in the first-mentioned project and the Structured Clinical Interview for DSM-5 Personality Disorders [42] in the latter project. Both projects were approved by the Regional Committee for Medical Research Ethics in Norway (REK-ID 2009/2016 and 2014/1696, respectively).

### Procedures non-clinical sample

The non-clinical sample was based on anonymous data from 467 persons recruited as healthy controls for the Norwegian “Thematically Organized Psychosis Research Study” [43]. These participants were aged 18–65 and were randomly selected from the population registry of Oslo and screened for the presence of intellectual disability, mental illness, neurological illness, head trauma, substance misuse in the last year, and severe mental illness in first-degree relatives. A matched extraction from this group, based on gender and age, was conducted for the current study (*n* = 157).

### Measures

Multiple measures of TTM and SPD severity were employed to capture complementary perspectives on symptom severity, encompassing both clinician-rated and self-reported assessments.

### The national institute of mental health trichotillomania severity scale (NIMH-TSS) [44]

The NIMH-TSS is a clinician-rated instrument designed to assess the severity of TTM. Five core dimensions are evaluated: the amount of time spent pulling hair over the past week; time spent on the most recent day; urge resistance, subjective distress caused by pulling; and the extent to which pulling interferes with daily functioning. Each dimension is rated on a 6-point Likert scale from 0 to 5, yielding a total score between 0 and 25. Higher scores indicate greater TTM symptom severity.

The NIMH-TSS was also adapted for assessing skin-picking severity in patients with SPD by replacing the term “pulling” with “picking”. For TTM, the NIMH-TSS has demonstrated adequate convergent and discriminant validity, although test-retest reliability and the internal consistency (e.g., α ≈ 0.52) has been reported as low [45]. To our knowledge, the psychometric properties of the SPD-adapted NIMH-TSS have not been evaluated. In the current study, Cronbach’s alpha was 0.62 in the TTM group and 0.54 in the SPD group.

### Clinical global impression scale (CGI-S) [46]

The CGI-S is a clinician-rated scale that assesses the global severity of the illness on a scale from 1 (*“normal”*) to 7 (*“among the most severely ill patients”*). The measure has demonstrated good psychometric properties (e.g., good inter-rater reliability and validity) across several mental disorders [47, 48], including TTM [49]. In the present study, experienced clinicians used a CGI-S scale modified for TTM and SPD [33].

### The massachusetts general hospital hairpulling scale (MGH-HPS) [50]

The MGH-HPS is a 7-item self-report instrument measuring the severity of hair-pulling during the past week. The first three items assess the frequency, intensity, and perceived control over the urge to pull hair. The last four items assess the frequency of and distress related to hair-pulling, as well as attempts to resist the behavior and the degree of success of those attempts. Each item is rated on a 5-point Likert-scale ranging from 0 (*“low severity”*) to 4 (*“high severity”*). The total score ranges from 0 to 28, with higher scores indicating a greater severity of hair-pulling. The MGH-HPS has shown good internal consistency (*α* = 0.83) and construct validity, but the test-retest reliability is somewhat low [45, 51]. In the current study, Cronbach’s alpha was 0.89 in the TTM group.

### Skin picking scale-revised (SPS-R) [52]

The SPS-R is an 8-item self-report measure assessing the severity of skin-picking during the past week. The first four items assess the frequency and intensity of the urge to pick, the time spent picking, as well as the perceived control over skin-picking. The last four items assess the degree of distress, functional impairment, avoidance, and the damage related to skin-picking [53]. Each item is rated on a 5-point Likert scale ranging from 0 (*“no behavior/experience”*) to 4 (*extreme behavior/experience”*), resulting in a total score between 0 and 32. Higher scores indicate more severe skin-picking symptoms. The psychometric properties for SPS-R have been shown to be good, and it is a reliable and valid tool for assessing the severity of skin-picking [52]. In the current study, Cronbach’s alpha was 0.78 in the SPD group.

### Childhood trauma questionnaire – short form (CTQ-SF) [20]

The CTQ-SF is a 25-item self-report instrument that assesses five subtypes of childhood maltreatment: (1) physical abuse, (2) sexual abuse, (3) emotional abuse, (4) physical neglect, and (5) emotional neglect [27]. Each subscale consists of five items, where each item is rated on a 5-point Likert scale from 1 (*“not right”*) to 5 *(“very often true”*). The total score ranges from 25 to 125, with higher scores reflecting more severe child abuse and neglect. The CTQ-SF is one of the most widely used instruments to measure childhood maltreatment and has shown strong psychometric properties [54], also in Norwegian samples [55]. In our four samples, the physical neglect subscale stood out by showing low Cronbach’s alpha values (range: 0.50 − 0.59), which is consistent with previous research [54]. For the remaining four subscales, Cronbach’s alpha values were within the acceptable range across groups: TTM: 0.80 − 0.90; SPD: 0.81 − 0.94; BPD: 0.87 − 0.93; healthy controls: 0.75 − 0.89.

### Patient health questionnaire (PHQ-9) [56]

The PHQ-9 is a 9-item self-report inventory measuring symptoms of depression. Each item is rated on a 4-point Likert scale from 0 (*“not at all”*) to 3 (*“nearly every day”*), resulting in a total score between 0 and 27. Higher scores indicate more severe depressive symptoms. A total score between 4 and 9 is commonly interpreted as mild severity, 10–14 as moderate, and 15–21 as severe [57]. The psychometric properties of PHQ-9 are well established across different settings (e.g., clinical settings and in the general population) and countries [57–59]. In the current study, Cronbach’s alpha was 0.88 in the TTM group and 0.86 in the SPD group.

### Data analysis

The statistical analyses were conducted using the IBM SPSS Statistics version 29 and STATA version 17. Prior to the analysis, the datafile was reviewed to check for missing values in CTQ-SF. 35 out of 3925 items (0.89%) single CTQ-SF items were missing in the TTM/SPD sample, and 9 out of 3925 items (0.23%) in the healthy controls. Visual inspection and skewness analysis of CTQ-SF, PHQ-9, MGH-HPS, and SPS-R indicated acceptable skewness levels for MGH-HPS (-0.41), SPS-R (0.21), and PHQ-9 (0.13). Some CTQ-SF subscales had high skewness levels, e.g., physical abuse (3.26) and sexual abuse (3.20). Non-parametric tests were therefore used in the statistical analyses where CTQ-SF was involved.

Healthy controls were matched with the TTM and SPD patients based on age and gender. Matching BPD patients with TTM and SPD patients was possible for age and male gender but not for female gender due to a smaller number of women in the BPD sample than the TTM and SPD sample. However, the age distribution was very similar in these three groups (see Table 1).

Demographic and clinical characteristics of the four groups (TTM, SPD, BPD, and the healthy controls) were compared using chi-square test for dichotomous variables and t-tests or one-way analysis of variance (ANOVA) for continuous variables. Given significant results from Levene´s test for unequal variances, the Games-Howell post hoc test was used, which is considered robust handling unequal variances [60]. Effect sizes were calculated using partial eta-squared (*η*^*2*^_*p*_), with a value of 0.01 indicating small effect, 0.06 a medium effect, and 0.14 a large effect, according to Cohen´s *d*. Kruskal-Wallis tests with Bonferroni-corrected Dunn’s post hoc tests were conducted instead of ANOVA in the analyses involving CTQ-SF.

To explore the relationship between TTM and SPD symptom severity, CM, and symptoms of depression, Spearman´s bivariate correlation coefficients were calculated among the scores of MGH-HPS, SPS-R, NIMH-TSS, CGI-S, CTQ-SF, and the PHQ-9. A correlation coefficient < 0.30 is considered small, 0.30 − 0.50 as moderate, and > 0.50 as strong [61].

To explore whether depressive symptoms (PHQ-9) mediated the relationship between emotional neglect/emotional abuse (subscales of CTQ-SF) and TTM severity (MGH-HPS and NIMH-TSS), four mediation analyses were performed in STATA with the sem-package. To address the violation of the normality assumption in the CTQ-SF data, a bootstrapping procedure was employed to estimate the indirect effect, with 95% confidence intervals calculated [62]. A total of 10.000 bootstrap samples were generated. The NIMH-TSS was selected over the CGI-S as the clinician-rated outcome measure in the mediation analyses because it provides a more detailed and disorder-specific assessment of TTM symptom severity. In contrast to the CGI-S, which is a global measure of clinical severity, the NIMH-TSS captures multiple dimensions of hair-pulling behavior and is one of the few clinician-administered instruments specifically developed for TTM [45].

## Results

### Participant characteristics

The demographic and clinical characteristics of the TTM, SPD, and BPD patients, along with those of the healthy control group, are shown in Table 1. All four groups had a majority of women (89.5–93.3%), and there were no significant differences in gender or age between the groups.

**Table 1:** Comparison of sociodemographic and clinical participant characteristics: TTM vs. SPD vs. BPD vs. healthy control group

	TTM	SPD	BPD	Control			
	(*n* = 82)	(*n* = 75)	(*n* = 123)	(*n* = 157)			
Characteristics	*%*	*%*	*%*	*%*	*x* ^*2*^	*Phi*	*Post hoc*
Female gender	90.2	93.3	89.5	91.7	0.49	0.04	
Employed/student	80.2^a^	84.0	41.4^e^	-	30.1**	0.36	BPD > TTM**, BPD > SPD**
Married/cohabitant	48.8	36.5^b^	38.6^e^	-	2.93	0.11	
Any comorbid mental disorder	79.3	61.3	93.2^e^	-	17.8**	0.29	BPD > TTM*, BPD > SPD**, TTM > SPD*
Major depressive disorder	55.8	34.8	60.4	-	10.96**	0.21	BPD > SPD**, TTM > SPD*
Dysthymia	16.9	6.1	25.0	-	9.87**	0.20	BPD > SPD**, TTM > SPD*
Bipolar disorder	5.2	4.5	9.6	-	2.00	0.09	
Anxiety disorder	66.2	50.0	60.0	-	3.92	0.14	
OCD	14.3	27.3	9.5	-	9.44**	0.20	SPD > BPD**
PTSD	13.2	9.2	18.2	-	2.68	0.11	
	*M (SD)*	*M (SD)*	*M (SD)*	*M (SD)*	*F or t*	*η* ^*2*^_*p*_ *or d*	*Post hoc*
Age	31.7 (11.7)	29.3 (7.7)	30.8 (8.2)	29.7 (7.8)	1.67	0.01	
Clinical Global Impression	5.0 (0.7)^c^	4.8 (0.7)^d^	-	-	2.05*	0.34	TTM > SPD
PHQ-9 total	11.6 (6.7)^a^	12.5 (6.1)^b^	19.0 (6.1)^f^	-	28.90**	0.21	BPD > TTM**, BPD > SPD**

*TTM*  Trichotillomania, *SPD* Skin-picking disorder, *BPD*  Borderline personality disorder, *Anxiety disorders* generalized anxiety disorder, panic disorder, agoraphobia, specific phobia, social anxiety disorder, *OCD* Obsessive-compulsive disorder, *PTSD* Post-traumatic stress disorder,* PHQ-9* Patient Health Questionnaire

*n* = 81; ^b^ *n* = 74; ^c^ *n* = 77; ^d=^*n*=70; ^e^ *n* = 59; ^f^ *n* = 67. ^e, f^= Only BPD patients from the Alternative Model for Personality Disorder study.

*p < .05, **p < .01.

Information on employment status, marital status, and psychiatric comorbidity was available for the TTM and SPD patients, as well as the largest subgroup of BPD patients (from the study on the Alternative Model for Personality Disorders). There were no significant group differences in marital status, as nearly half of the participants in all groups were married or cohabited. However, the BPD group had a significantly lower employment rate than the TTM and SPD groups (41.4% versus 80.2% and 84.0%). All patient groups reported high levels of psychiatric comorbidity, with major depressive disorder and anxiety disorders as the most common. The BPD and TTM group exhibited significantly higher rates of major depressive disorder and dysthymia compared to the SPD group, whereas the SPD group showed a significantly higher prevalence of obsessive-compulsive disorder than the BPD group.

The presence of BFRBs was not assessed in the BPD sample, and conversely, BPD was not assessed in the BFRB samples. Therefore, the proportion of BPD patients with comorbid TTM or SPD, as well as the proportion of TTM/SPD patients with comorbid BPD, remains unknown. The proportion of self-identified comorbid skin-picking in patients with TTM was 17.9%, whereas 16.4% of patients with SPD reported self-identified hair-pulling problems.

The mean baseline MGH-HPS score among the TTM patients was 17.2 (*SD* = 5.1), whereas the mean baseline SPS-R score of the SPD patients was 17.5 (*SD* = 3.9). The TTM group had a slightly higher CGI-S score than the SPD group; however, the effect size was small. The BPD group reported significantly more severe depressive symptoms, as measured by the PHQ-9, than both the SPD and TTM groups, while no difference emerged between the SPD and TTM groups. All groups had a mean PHQ-9 score above the clinical threshold of 10, indicating at least moderate severity of depressive symptoms.

### Comparison of CM between patients with TTM, SPD and BPD, and healthy controls

Table 2 presents the mean CTQ-SF scores for patients with TTM, SPD, and BPD prior to treatment, as well as the CTQ-SF scores of the healthy control group. As expected, the Kruskal-Wallis test results revealed significant group differences in the total CTQ-SF score and across all five CM subscales. The largest group differences were observed for emotional abuse and emotional neglect.

**Table 2:** Comparison of childhood maltreatment among individuals with TTM, SPD, BPD, and healthy controls

	TTM (*n* = 82)	SPD (*n* = 75)	BPD (*n* = 123)	Controls (*n* = 157)			
Schema	M (SD)	M (SD)	M (SD)	M (SD)	KWH	*p*	Post-hoc
Emotional abuse	0.72 (0.87)	1.00 (0.99)	1.76 (1.12)	0.29 (0.45)	146.65	< 0.001	BPD > TTM, SPD > controls
Physical abuse	0.23 (0.58)	0.18 (0.51)	0.63 (0.95)	0.05 (0.26)	68.72	< 0.001	BPD > TTM > controls; BPD > SPD
Sexual abuse	0.26 (0.68)	0.43 (0.76)	0.68 (1.12)	0.07 (0.29)	66.99	< 0.001	BPD > TTM > controls; SPD > controls
Emotional neglect	1.17 (1.08)	1.24 (1.17)	2.33 (1.03)	0.47 (0.63)	167.75	< 0.001	BPD > TTM, SPD > controls
Physical neglect	0.34 (0.53)	0.37 (0.54)	0.86 (0.81)	0.17 (0.37)	91.31	< 0.001	BPD > TTM, SPD > controls
CTQ-SF total	0.54 (0.60)	0.64 (0.63)	1.25 (0.77)	0.21 (0.31)	178.43	< 0.001	BPD > TTM, SPD > controls

*TTM* Trichotillomania, *SPD* Skin-picking disorder, *BPD* Borderline personality disorder, *KWH* Kruskal_Wallis H, *CTQ-SF* Childhood Trauma Questionnaire Short-Form

No significant differences were found between the TTM and SPD groups. However, both patient groups reported significantly higher levels of CM than the control group across all subscales, except that the SPD group did not differ from controls on physical abuse. In contrast, when compared to individuals with BPD, patients with TTM and SPD scored significantly lower on the total CTQ-SF score and all subscales, except for sexual abuse, where BPD and SPD patients reported similar levels.

To examine whether comorbidity between TTM and SPD influenced the results, the Kruskal-Wallis tests were rerun after excluding 14 TTM patients with a self-identified skin-picking problem and 12 SPD patients with a self-identified hair-pulling problem. The results of these analyses are presented in Table A1 in Supplementary Materials. Overall, the findings were consistent with the original results. However, the difference between the TTM group and healthy controls was no longer significant for sexual abuse and physical neglect.

To examine potential differences between subthreshold BPD and threshold BPD, we conducted a series of sensitivity analyses (independent sample t-tests) comparing mean scores across the five CTQ subscales and the total CTQ score. No significant differences were observed; the smallest p-value was 0.301 (emotional abuse).

### Correlations between CM and symptom severity of TTM and SPD

Correlations between CM and symptom severity of TTM and SPD are presented in Table 3. As hypothesized, both clinician-rated (i.e., only NIMH-TSS) and self-reported (MGH-HPS) measures of TTM severity showed significant positive correlations with the CTQ-SF total score and most of its subscales. These correlations ranged from mild to moderate in strength (*r* = .22 − .41). Notably, emotional abuse was the CTQ-SF subscale most strongly associated with both self-reported and clinician-rated TTM severity (NIMH-TSS and CGI-S-TTM).

**Table 3:** Spearman’s correlations between TTM and SPD severity, CM, and depressive symptoms

	Schema	1 (*n* = 81)	2 (*n* = 78)	3 (*n* = 78)	4 (*n* = 74)	5 (*n* = 67)	6 (*n* = 70)	7 (*n* = 155)
TTM severity	1. MGH-HPS	-						
	2. NIMH-TSS: TTM	0.34**	-					
	3. CGI-S – TTM	0.24*	0.57**	-				
SPD severity	4. SPS-R	-	-	-	-			
	5. NIMH-TSS: SPD	-	-	-	0.57**	-		
	6. CGI-S – SPD	-	-	-	0.24	0.23	-	
Symptoms of depression	7. PHQ-9	0.38**	0.33**	0.19	0.51**	0.13	0.31**	-
Childhood maltreatment	8. CTQ-SF total	0.28*	0.31**	0.19	0.06	0.05	0.27*	0.36**
	9. Emotional abuse	0.34**	0.41**	0.36**	0.08	0.05	0.34**	0.40**
	10. Physical abuse	0.29**	0.15	0.14	0.15	0.11	0.20	0.13
	11. Sexual abuse	0.33*	0.13	0.15	0.07	0.15	0.33**	0.21**
	12. Emotional neglect	0.22**	0.26*	0.10	0.05	− 0.02	0.11	0.28**
	13. Physical neglect	0.16	0.26*	0.20	0.13	0.01	0.15	0.34**

*TTM*  Trichotillomania, *SPD* Skin-picking disorder, *CM*  Childhood maltreatment, *MGH-HPS* Massachusetts Gen. Hospital Hairpulling Scale, *NIMH-TSS* National Institute of Mental Health Trichotillomania Severity Scale, *SPS-R* Skin Picking Scale Revised, *CGI-S *Clinical Global Impression Scale - Severity, *PHQ-9 *Patient Health Questionnaire, *CTQ-SF *Childhood Trauma Questionnaire Short-Form

*p < .05, **p < .01

There were no significant associations between self-reported SPD severity (SPS-R) and CTQ-SF, neither total score nor subscales. The results were mixed regarding clinician-rated SPD severity. No significant correlations were found between CTQ-SF and NIMH-TSS. However, three significantly positive associations emerged between CGI-S-SPD and CTQ-SF: CTQ-SF total score (*r* = .27), emotional abuse (*r* = .34), and sexual abuse (*r* = .33). These correlations were of mild to moderate strength.

### Mediation analysis

In line with the research questions, four mediation analyses were conducted in the TTM sample to investigate whether depressive symptoms (PHQ-9) would mediate the association between emotional neglect/emotional abuse (CTQ-SF) and TTM severity (MGH-HPS and NIMH-TSS). As shown in Table 3, both predictors were significantly correlated with the potential mediator, and all three variables were significantly correlated with the outcome variables (TTM severity). This pattern of association fulfilled the criteria necessary for mediation [62].

**Fig. 1:**
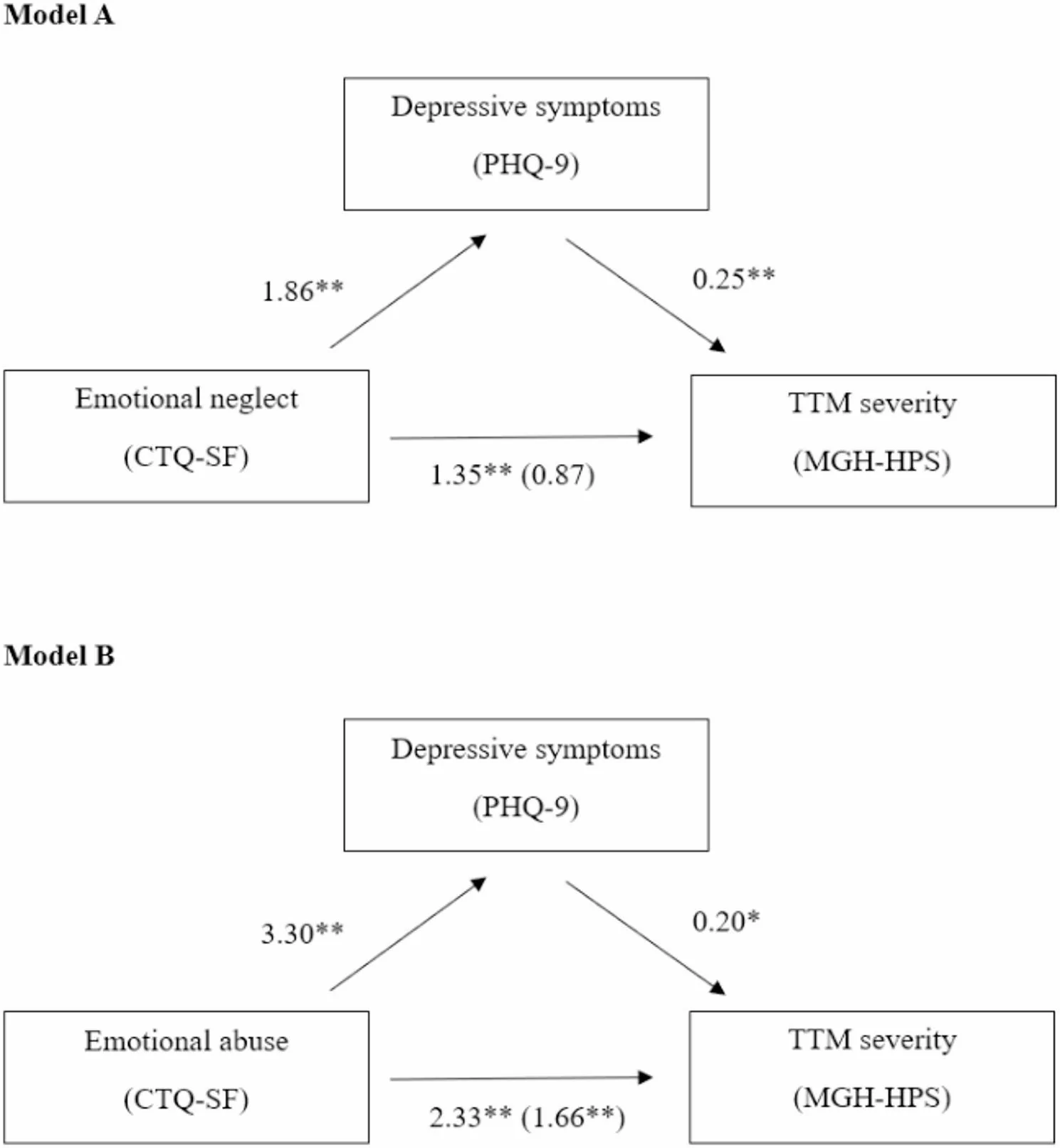
Mediation results in the TTM sample. Note. TTM = trichotillomania; CTQ-SF = Childhood Trauma Questionnaire Short-Form; PHQ-9 = Patient Health Questionnaire; MGH-HPS = Massachusetts General Hospital Hairpulling Scale. *p <.05, **p <.01

As illustrated in Fig. 1 (Model A), the direct effect of emotional neglect on self-reported hair-pulling severity was significant (*B* = 1.35, SE = 0.52, *p* = .010). When depressive symptoms were entered as a mediator, the regression coefficient between emotional neglect and self-reported TTM severity became non-significant (*B* = 0.87, SE = 0.49, *p* = .075). As such, the results indicated that depressive symptoms fully mediated the relationship between emotional neglect and TTM severity. A proportion of 35.1% of the total effect was mediated by depressive symptoms.

As illustrated in Fig. 1 (Model B), the direct effect of emotional abuse on self-reported hair-pulling severity was significant (*B* = 2.33, SE = 0.51, *p* < .001). When depressive symptoms were included in the model, the regression coefficient between emotional neglect and TTM severity became notably smaller, but was still significant (*B* = 1.66, SE = 0.48, *p* = .001). The results therefore indicated that depressive symptoms partially mediated the relationship between emotional abuse and TTM severity. Depressive symptoms accounted for 28.8% of this relationship.

In the two mediation analyses examining whether depressive symptoms mediated the relationship between emotional abuse/neglect and clinician-rated TTM severity (Model C and D with NIMH-TSS as outcome variable), neither analysis showed a statistically significant mediation effect. Specifically, both path b and the indirect effect were non-significant. A summary of the total, direct, and indirect effects, along with the estimated path coefficients for all four mediation analyses, is presented in Table 4.

**Table 4:** Total, direct, and indirect effects in the mediation models

Model	Effect	B	SE	95% Bootstrap CI	*p*
Model A	Total effect (C)	1.35	0.50	[0.37, 2.32]	0.007
	Direct effect (c’)	0.87	0.48	[-0.06,1.81]	0.068
	Indirect effect (a * b)	0.47	0.21	[0.06, 0.88]	-
	Path a (EN → PHQ-9)	1.86	0.54	[0.80, 2.92]	0.001
	Path b (PHQ-9 → MGH-HPS)	0.25	0.08	[0.10, 0.41]	0.001
Model B	Total effect (C)	2.33	0.50	[1.34, 3.31]	< 0.001
	Direct effect (c’)	1.66	0.48	[0.71, 2.60]	0.001
	Indirect effect (a * b)	0.67	0.31	[0.07, 1.27]	-
	Path a (EA → PHQ-9)	3.30	0.70	[1.93, 4.66]	< 0.001
	Path b (PHQ-9 → MGH-HPS)	0.20	0.08	[0.05, 0.36]	0.011
Model C	Total effect (C)	1.13	0.45	[0.25, 2.01]	0.012
	Direct effect (c’)	0.85	0.46	[-0.06, 1.76]	0.067
	Indirect effect (a * b)	0.29	0.16	[-0.02, 0.59]	-
	Path a (EN → PHQ-9)	2.03	0.55	[0.96, 3.10]	< 0.001
	Path b (PHQ-9 → NIMH-TSS)	0.14	0.07	[-0.00, 0.28]	0.052
Model D	Total effect (C)	2.05	0.40	[1.28, 2.83]	< 0.001
	Direct effect (c’)	1.74	0.45	[0.86, 2.62]	> 0.001
	Indirect effect (a * b)	0.31	0.28	[-0.24, 0.86]	-
	Path a (EA → PHQ-9)	3.60	0.78	[2.07, 5.13]	> 0.001
	Path b (PHQ-9 → NIMH-TSS)	0.09	0.08	[-0.06, 0.23]	0.254

Unstandardized coefficients are reported. Bootstrap confidence intervals are based on 10.000 resamples.

*EN *Emotional neglect, *EA* Emotional abuse, *PHQ-9* Patient Health Questionnaire, *MGH-HPS *Massachusetts Gen. Hospital Hairpulling Scale

There was no significant association between emotional neglect/abuse and SPD severity, as measured with SPS-R and NIMH-TSS. Mediational analyses of the relationship between emotional neglect/emotional abuse, depressive symptoms, and SPD severity were therefore not conducted.

## Discussion

This study aimed to explore the relationship between self-reported CM and severity of TTM and SPD, as well as comparing TTM and SPD patients’ CM severity to a group of BPD patients and healthy controls. As hypothesized, the TTM and SPD patients reported higher levels of CM (especially emotional abuse and emotional neglect) than the healthy controls, but lower levels than the BPD patients. Several forms of CM were significantly positively associated with self-reported and clinician-rated TTM and SPD severity, although the associations were more numerous and stronger in TTM patients than in SPD patients. Depressive symptoms mediated the relationship between emotional abuse/emotional neglect, and self-reported TTM severity. These results are consistent with, though do not prove, an emotion-regulation model in which hair-pulling serves as a maladaptive coping strategi for depressive symptoms.

### Comparison of CM among patients with TTM, SPD, BPD, and healthy controls

Our results are consistent with previous literature on CM in TTM and SPD [17–19], suggesting that CM is common among individuals with TTM and SPD. The results are also consistent with previous literature finding elevated levels of CM in other mental disorders such as depression, anxiety, and personality disorders, thereby corroborating CM as a well-known risk factor for adult mental disorders [14–16].

The reanalysis of the Kruskal–Wallis tests excluding participants with comorbid hair-pulling and skin-picking problems yielded results largely consistent with the original analyses. However, the difference between the TTM group and healthy controls was no longer significant for sexual abuse and physical neglect. This change may reflect reduced statistical power due to the smaller sample size, but it may also suggest that comorbid cases contributed to the group differences observed in the original analyses.

Patients with TTM and SPD scored significantly lower than patients with BPD on all but one form of CM. This finding was expected, as a previous meta-analysis of childhood adversity and BPD [24] found individuals with BPD to be 3.15 times more likely to report childhood adversity than other psychiatric groups, such as mood disorders, psychosis, and other personality disorders. The current study was however the first to show that this finding also applies to the comparison between BPD and TTM/SPD. Porter et al. [24] suggested that the elevated prevalence of childhood adversity among individuals diagnosed with BPD might be attributed to an overlap between the diagnostic criteria of complex PTSD and BPD, as well as the high rates of comorbidity commonly observed in BPD populations. Consequently, the findings might reflect an increased burden of comorbid psychopathology associated with childhood trauma, rather than being specific to BPD itself.

Even though the between-group differences were most pronounced for emotional abuse and emotional neglect, these two forms of CM stood out as having the highest severity across all four groups – including healthy controls. These two types of CM have been found to be among the most common in both clinical and non-clinical populations [19, 55, 63].

### Relationship between CM and TTM severity

As hypothesized, self-reported and clinician-rated TTM severity (i.e., MGH-HPS and NIMH-TSS) were significantly positively correlated with the severity of several types of CM, with the strongest associations for emotional abuse (*r*: 0.34 − 0.41). The mediator analysis showed that depressive symptoms fully mediated the relationship between emotional neglect and self-reported hair-pulling severity and partially mediated the relationship between emotional abuse and self-reported hair-pulling severity. This is consistent with the finding of Houghton et al. [27], although they used different variables for trauma (i.e., dichotomous variable: presence of trauma), symptoms of depression (BDI), and hair-pulling severity (CGI-S). These findings support, though do not prove, the indirect link proposed by Houghton et al. [27], where traumatic experiences are supposed to lead to negative affect (e.g., depressive symptoms), followed by hair-pulling being used as a maladaptive mechanism to regulate this negative affect. They also align with existing literature suggesting that hair-pulling can be used to regulate internal states [28, 64–67].

However, depressive symptoms did not mediate the relationship between emotional abuse/neglect and clinician-rated TTM severity (NIMH-TSS). One possible explanation for this finding is the mixed psychometric properties of the NIMH-TSS. In a recent psychometric evaluation (*N* = 91), Barber et al. [45] reported low internal consistency (α = 0.52) and low test–retest reliability (*r* = .61) for the scale and found that NIMH-TSS scores were not associated with objective indices of hair-loss severity. This pattern suggests that the NIMH-TSS may capture heterogeneous aspects of TTM symptoms that are not strongly interrelated (e.g., time spent on hair-pulling versus impact of hair-pulling), potentially limiting its sensitivity to detect associations with mediating variables such as depressive symptoms. Consistent with this, relatively low internal consistency was also observed in the present study (α = 0.62 in the TTM sample), which may have attenuated associations and reduced the likelihood of detecting a significant indirect effect.

### Relationship between CM and SPD severity

Fewer significant associations between CM and SPD severity were identified than expected. No significant correlations were found between CM and self-reported SPD severity (SPS-R), and only a few significant associations emerged between clinician-rated SPD severity (CGI-S-SPD) and specific CM domains, namely emotional and sexual abuse. Thus, although individuals with SPD reported higher levels of CM than the healthy controls, the severity of these experiences does not appear to be related to the severity of SPD symptoms.

The discrepancy in the strength of the association between CM and TTM versus SPD severity may reflect a group difference between these two disorders, with CM playing a more prominent role in TTM than in SPD. Although the disorders share several demographic and phenomenological similarities, group differences have also been reported [68, 69]. Specifically, Snorrason et al. [69] outlined several potential etiological differences between TTM and SPD, suggesting that dermatological problems and concerns about physical appearance may constitute stronger risk factors for SPD than for TTM. Thus, the weaker association between CM and SPD severity observed in our study may point to differences in etiological pathways across SPD and TTM. That is, SPD might be more strongly associated with dermatological conditions, whereas TTM appears to be more strongly associated with emotional neglect and emotional abuse. Differences in etiological pathways are further corroborated by family-history studies have shown that relatives of individuals with TTM are more likely to have TTM than SPD, whereas relatives of individuals with SPD are more likely to have SPD than TTM. Based on these findings, Snorrason et al. [69] argued that there is a need to examine familial environmental factors or genetic mechanisms that may differentially influence the risk for TTM and SPD.

The findings may also be attributable to methodological factors, particularly assessment-related bias. Both the SPS-R and NIMH-TSS are based on BFRB symptoms during the last week. Thus, the small and non-significant correlations between CM and these measures could be explained by larger degree of fluctuations of BFRB symptoms in the SPD group than in the TTM group. The CGI-S, on the other hand, is based on a global impression of SPD severity and might be less sensitive to contemporary fluctuations of SPD symptoms, which may explain why this measure showed several significant correlations with CM.

### Strengths and limitations

The current study holds several strengths, such as the large, clinical sample of individuals with TTM and SPD, and the use of a healthy control group and a clinical group of BPD patients matched by age and gender. The severity of TTM and SPD symptoms observed in our sample were in line with those reported in previous clinical studies [70, 71], which supports the representativeness of our sample and increases the generalizability of our findings. However, several limitations need to be taken into consideration.

First, our TTM and SPD samples had a strong predominance of women, suggesting that the results may be less generalizable to men with these disorders. The participants were also treatment-seeking, which may have further skewed the gender distribution, as women are more likely to seek treatment [72].

Second, because the samples were drawn from four different research protocols, differences in recruitment procedures, assessment contexts, and inclusion criteria may have introduced heterogeneity that could have affected the findings. Moreover, the presence of BFRBs was not evaluated within the BPD sample, nor was BPD systematically assessed in the BFRB samples, preventing us from controlling for potential comorbidity. Given previous findings indicating that BFRBs are characterized by emotional dysregulation and impulsivity [28, 69], it is not unlikely that there is a considerable overlap between BFRBs and BPD. Indeed, an online survey found that 281 patients with BFRBs screened positive for a probable BPD diagnosis (37%) [73]. Although screening instruments are prone to elevated false-positive rates, these findings nonetheless highlight the potential for substantial comorbidity between the two conditions.

Third, the use of self-report measures to correlate CM, depressive symptoms, and TTM/SPD severity can be a limitation, as the correlation estimates might be inflated due to shared method variance and self-reporting biases [74]. The use of a self-report measure might also increase the risk of recall bias as well as implicit theory bias [75], e.g., participants may vary in how they define or interpret what qualifies as a traumatic experience. In addition, the clinician-rated NIMH-TSS adapted for SPD has not yet been psychometrically validated, which should be taken into account when interpreting findings involving clinician-rated SPD severity. The internal consistency of the NIMH-TSS was low in both groups, which may indicate that the measure is not a unitary construct, but rather captures different aspects of TTM severity. Further research is needed to examine its psychometric properties in both TTM and SPD samples, including the use of factor analysis.

Lastly, the cross-sectional design limits the ability to determine causality. Prospective research is necessary to investigate whether CM lead to more severe symptoms of TTM and SPD.

## Conclusion

These findings suggest that individuals with TTM and SPD have higher levels of CM as compared with healthy controls. The results are also consistent with, though do not prove, an emotion-regulation model in which hair-pulling serves as a maladaptive coping strategi for depressive symptoms. The lack of a significant correlation between CM and self-reported SPD severity may be attributable to methodological factors such as assessment-related bias. However, it may also reflect underlying differences in terms of etiological factors between the two patient groups (TTM vs. SPD). Specifically, CM may play a less prominent role in SPD than in TTM, as dermatological conditions may have a stronger influence in SPD.

The results emphasize the need for further clinical and prospective research to gain more knowledge about the association between CM and TTM/SPD severity (including the role of depressive symptoms as a potential mediator), and to explore the differences and similarities in phenomenology and clinical presentation between individuals with TTM and SPD. This would be important to develop effective interventions for individuals suffering from these disorders.

## Supplementary Information

Supplementary Material 1.

## Data Availability

Due to restrictions imposed by the Regional Medical Ethics Committee to protect patient confidentiality, the data are available only upon reasonable request. Requests should be directed to the Privacy and Data Protection Officer at Oslo University Hospital, Oslo, Norway, [personvern@ous-hf.no] (mailto: personvern@ous-hf.no) .
