# Supplementary material for: Trichotillomania and skin-picking disorder: associations with childhood maltreatment and the mediating role of depressive symptoms
Source: BMC Psychol. 2026 Jul 20;14:1481. doi: 10.1186/s40359-026-05193-y (PMC13670518; doi:10.1186/s40359-026-05193-y)
Supplement: Supplementary file 1 [file 40359_2026_5193_MOESM1_ESM.docx]

|  | TTM  (*n*=68) | SPD  (*n*=63) | BPD  (*n*=123) | Controls  (*n*=157) |  |  |  |  |
| --- | --- | --- | --- | --- | --- | --- | --- | --- |
| Schema | *M (SD)* | *M (SD)* | *M (SD)* | *M (SD)* | *KWH* | *p* | Post-hoc |  |
| Emotional abuse | 0.67 (0.83) | 1.00 (1.03) | 1.76 (1.12) | 0.29 (0.45) | 144.17 | <.001 | BPD > TTM, SPD > controls |  |
| Physical abuse | 0.23 (0.54) | 0.20 (0.54) | 0.63 (0.95) | 0.05 (0.26) | 67.02 | <.001 | BPD > TTM > controls; BPD > SPD |  |
| Sexual abuse | 0.24 (0.69) | 0.42 (0.72) | 0.68 (1.12) | 0.07 (0.29) | 67.38 | <.001 | BPD > TTM; BPD > controls; SPD > controls | |
| Emotional neglect | 1.12 (1.05) | 1.21 (1.21) | 2.33 (1.03) | 0.47 (0.63) | 163.97 | <.001 | BPD > TTM, SPD > controls |  |
| Physical neglect | 0.31 (0.52) | 0.40 (0.58) | 0.86 (0.81) | 0.17 (0.37) | 91.51 | <.001 | BPD > TTM, SPD > controls |  |
| CTQ-SF total | 0.51 (0.58) | 0.65 (0.66) | 1.25 (0.77) | 0.21 (0.31) | 172.48 | <.001 | BPD > TTM, SPD > controls |  |

**Table A1**

*Comparison of Childhood Maltreatment among Individuals with TTM, SPD, BPD, and Healthy Controls*

*Note*. The Kruskal-Wallis tests were rerun after excluding 14 TTM patients with self-identified skin-picking problems and 12 SPD patients with a self-identified hair-pulling problem. TTM = trichotillomania; SPD = skin-picking disorder; BPD = borderline personality disorder; KWH = Kruskal_Wallis H; CTQ-SF = Childhood Trauma Questionnaire Short-Form.
